# Diosgenin Exerts Antitumor Activity via Downregulation of Skp2 in Breast Cancer Cells

**DOI:** 10.1155/2020/8072639

**Published:** 2020-06-16

**Authors:** Yanling Liu, Zijun Zhou, Jingzhe Yan, Xuefeng Wu, Guiying Xu

**Affiliations:** ^1^Department of Breast Medicine, Jilin Province Cancer Hospital, Changchun 130012, China; ^2^Department of Breast Surgery, Jilin Province Cancer Hospital, Changchun 130012, China; ^3^Department of Abdominal Oncosurgery, Jilin Province Cancer Hospital, Changchun, Jilin 130021, China; ^4^Department of Clinical Laboratory, Jilin Province Cancer Hospital, Changchun, Jilin 130012, China

## Abstract

**Background:**

Breast cancer is the common malignancy with high morbidity and mortality in women. S-phase kinase-associated protein 2 (Skp2) has been characterized to play an oncogenic role in the breast carcinogenesis and progression. Therefore, inactivation of Skp2 in breast cancer might be a novel approach for fighting breast malignancy. A natural compound diosgenin has been reported to exert anticancer activity in a variety of human cancers. However, the underlying mechanism has not been fully determined.

**Methods:**

In this study, we aim to explore whether diosgenin performed antitumor activity via inhibition of Skp2 in breast cancer cells using several methods including MTT, Transwell invasion assay, RT-PCR, western blotting, and transfection.

**Results:**

We found that diosgenin inhibited cell viability and stimulated apoptosis. Moreover, we found that diosgenin reduced cell invasion in breast cancer cells. Furthermore, diosgenin inhibited the expression of Skp2 in breast cancer cells. Notably, diosgenin reduced cell viability and motility and induced apoptosis via suppression of Skp2 in breast cancer cells.

**Conclusion:**

Our findings revealed that diosgenin could be a potential inhibitor of Skp2 for treating breast cancer.

## 1. Introduction

Breast cancer is the common malignancy in female and has an increasing incidence in the world. Patients with breast cancer have metastasis, leading to high morbidity and mortality in women [1]. More than 270,000 new cases of breast cancer are expected in the United States in 2020, which contributes to the most common cancer in the US [2]. Additionally, 42,170 deaths due to breast cancer will occur in America, leading to the second leading reason of cancer mortality after lung cancer [2]. The 5-year survival rate is 90% in the US [2]. Currently, the treatment approaches of breast cancer often have surgery, radiation, and chemotherapy. Although increased use of mammography screening and advanced therapeutic strategy have been applied for breast cancer, the patients with metastasis often have worse outcome and poor survival rate. Thus, it is required to discover the new agents for breast cancer therapy.

Diosgenin, a steroidal sapogenin obtained from fenugreek seed, has been known to perform anticancer function in a variety of human cancers [3]. For instance, diosgenin inhibited cell growth via induction of cell cycle arrest and mitochondria-mediated apoptotic death in cholangiocarcinoma [4]. A study uncovered that diosgenin effectively suppressed the viability of breast cancer cells [5]. Moreover, another study defined that diosgenin reduced the expression of fatty acid synthase via suppression of phosphorylation of Akt and mTOR and promotion of JNK phosphorylation in breast cancer cells with Her2 overexpression [6]. Furthermore, diosgenin inhibited cell proliferation and stimulated apoptosis and led to cell cycle arrest at G1 phase through reduction of pAkt expression and Akt kinase activity in breast cancer cells [7]. Specifically, diosgenin decreased the expression of NF-kappaB, Bcl-2, cyclin D1, Cdk-2, Cdk-4, survivin, and XIAP in breast cancer cells [7].

Since diosgenin exhibits no obvious toxicity in the normal breast epithelial cells, it is important to explore the mechanism by which diosgenin exerts anticancer activity in breast cancer [7]. To this end, in this study, we explored the biological function of diosgenin in breast cancer. We also determined how diosgenin has an inhibitory effect on breast cancer cells. S-phase kinase-associated protein 2 (Skp2), as an F-box protein, has been characterized to play a critical role in oncogenesis and tumor progression via targeting substrates for ubiquitination and degradation [8]. Overexpression of Skp2 has been reported in the majority of human cancers, including mammary malignancy [9]. Moreover, high expression of Skp2 is correlated with poor survival in human cancer patients [10]. Moreover, Skp2 plays an oncogenic role via promotion of cell proliferation, migratory and invasive activity, metastasis, and drug resistance in breast cancer [9]. Since Skp2 is an important oncoprotein in breast cancer development, we performed multiple experiments to determine whether diosgenin inhibits the expression of Skp2 in breast cancer cells. We found that diosgenin decreased the expression of Skp2 in breast cancer cells, suggesting that diosgenin could be a useful compound to treat breast cancer patients via targeting Skp2.

## 2. Materials and Methods

### 2.1. Cell Culture

Human MCF7 and MDA-MB-231 cells were bought from cell culture bank (Shanghai Institutes for Biological Sciences, Chinese Academy of Sciences). Cells were cultured in DMEM with 10% fetal bovine serum (FBS) and 1% penicillin/streptomycin in an atmosphere with 5% CO_2_ at 37°C.

### 2.2. Cell Viability Assay

MCF7 and MDA-MB-231 cells (5000 cells/well) were cultured in 96-well plates overnight. The cells were treated with different concentrations of diosgenin. After 48 h and 72 h of diosgenin treatment, MTT (3-(4,5-dimethylthiazol-2-yl)-2,5-diphenyltetrazolium bromide) assay was performed to measure cell proliferation as described previously [11].

### 2.3. Cell Apoptosis Measurement

Breast cancer cells were cultured in 6-well plates overnight. The cells were exposed to various doses of diosgenin for 72 hours, and cell apoptotic death was measured by Annexin V-FITC/PI Kit via flow cytometry as described previously [11].

### 2.4. Cell Invasion Measurement

Breast cancer cells were seeded in a top chamber of inserts with Matrigel, and FBS-free DMEM with various doses of diosgenin was added in the top chamber. The bottom chambers were filled with the complete DMEM. After 20 h, the cells of the bottom chambers were incubated with Calcein AM for 30 minutes. The invaded cells were photographed by a fluorescent microscope [12].

### 2.5. Real-Time Reverse Transcription PCR Assay

MCF7 and MDA-MB-231 cells were incubated in 6-well plates overnight. Cells were exposed to different concentrations of diosgenin for 72 h. The total RNA was extracted with Trizol, and RT-PCR was performed as described before [13].

### 2.6. Western Blotting Assay

MCF7 and MDA-MB-231 cells were incubated in 10 cm dishes overnight. Then, cells were treated with various doses of diosgenin for 72 h. The proteins were extracted from the treated cells using lysis buffer. The proteins were separated by SDS-PAGE, and western blotting assay was conducted as described before [11].

### 2.7. Transfection

MCF7 and MDA-MB-231 cells were incubated in 10 cm dishes and were transfected with Skp2 siRNA (GenePharma, Shanghai, China), control siRNA, and pcDNA3-myc-Skp2 plasmid (Addgene, Watertown, MA, USA) for 48 h using Lipofectamine 2000 following the manufacturer's protocol.

### 2.8. Statistical Analysis

The data were analyzed with analysis of variance (ANOVA) followed by Tukey's post hoc test to compare different groups. *p* < 0.05 was considered as significant.

## 3. Results

### 3.1. Diosgenin Suppresses Cell Viability

**Figure 1:**
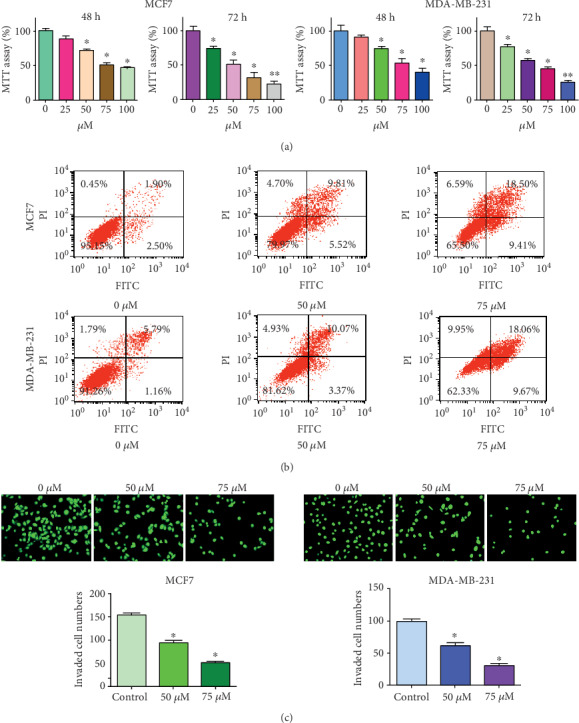
The effects of diosgenin on cell viability, apoptosis, and invasion. (a) MTT assay was used to test the cell viability in breast cancer cells after diosgenin treatment. ∗*p* < 0.05 and ∗∗*p* < 0.01 vs. the control group. (b) Annexin V-FITC/PI staining approach was used to test the cell apoptotic death in breast cancer cells after diosgenin treatment for 72 hours. (c) Top: a Transwell invasion assay was performed to test the invasive activity of breast cancer cells after diosgenin treatment for 20 hours; bottom: quantification data for the top panel. ∗*p* < 0.05 vs. the control group.

To test whether diosgenin could suppress the cell viability in breast cancer cells, we performed the MTT assay to measure viability of MCF7 and MDA-MB-231 cells after different doses of diosgenin treatment. Our MTT data showed that diosgenin significantly suppressed cell viability in both MCF7 and MDA-MB-231 cells (Figure 1(a)). The inhibitory effects on cell viability are shown in a dose-dependent manner in both breast cancer cell lines (Figure 1(a)).

### 3.2. Diosgenin Stimulates Cell Apoptosis

To determine whether diosgenin might regulate cell apoptotic death in breast cancer cells, we performed Annexin V-FITC/PI approach to examine the cell apoptosis in breast cancer cells after different concentrations of diosgenin treatment. Our results revealed that diosgenin stimulated cell apoptosis in both MCF-7 and MDA-MB-231 cells (Figure 1(b)). Moreover, diosgenin induced cell apoptosis in a dose-dependent manner in both breast cancer cell lines (Figure 1(b)).

### 3.3. Diosgenin Reduces Cell Invasion

To examine whether diosgenin might affect the cell motility on breast cancer cells, we measured cell invasive activity in MCF-7 and MDA-MB-231 cells after various doses of diosgenin treatments. Our experimental data demonstrated that diosgenin reduced cell invasive activity in both breast cancer cell lines in a dose-dependent manner (Figure 1(c)).

### 3.4. Diosgenin Reduces the Expression of Skp2

**Figure 2:**
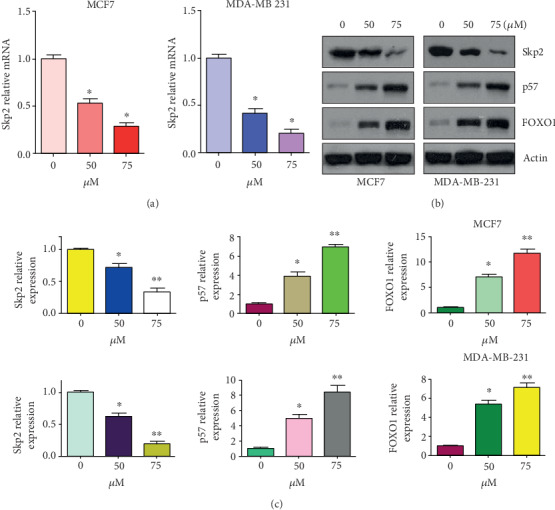
Diosgenin downregulates Skp2 expression. (a) The RT-PCR was used to measure the Skp2 mRNA level in breast cancer cells after diosgenin treatment for 72 hours. (b) The western blotting analysis was used to examine the expression of Skp2, p57, and FOXO1 in breast cancer cells after diosgenin treatment for 72 hours. (c) Quantification data for (b). ∗*p* < 0.05 and ∗∗*p* < 0.01 vs. the control group.

Since Skp2 is a key oncoprotein in human breast cancer, we tested whether diosgenin would downregulate the expression of Skp2 in breast cancer cells by RT-PCR and western blotting analysis. We found that diosgenin reduced the expression of Skp2 in MCF-7 and MDA-MB-231 cells at both mRNA and protein levels after diosgenin treatments for 72 hours (Figures 2(a)–2(c)). To confirm this data, we also measured the expression of Skp2 downstream targets, including p57 and FOXO1, in breast cancer cells treated with various doses of diosgenin for 72 hours. Our western blotting result showed that diosgenin upregulated the expression of p57 and FOXO1 in MCF-7 and MDA-MB-231 cells (Figures 2(b) and 2(c)).

### 3.5. Upregulation of Skp2 Abrogated Diosgenin-Mediated Anticancer Activity

**Figure 3:**
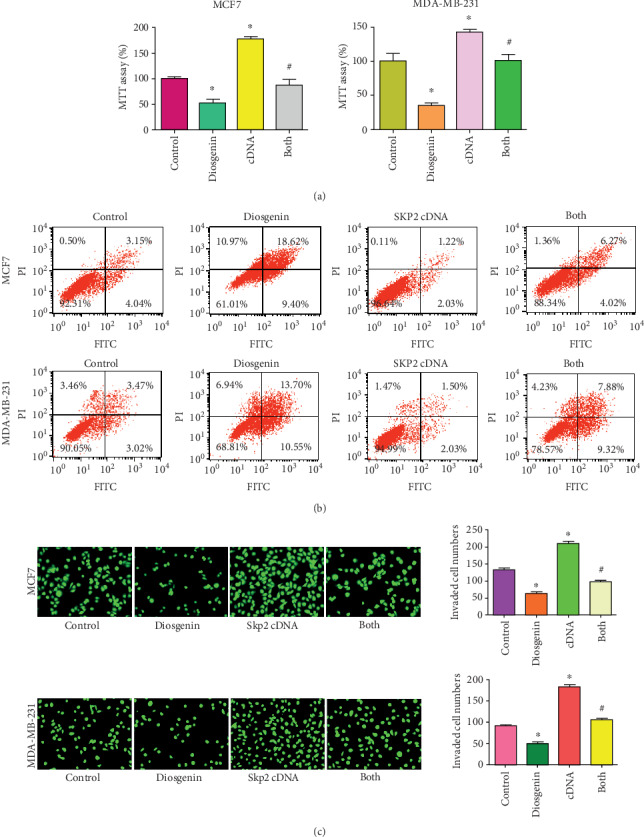
Skp2 upregulation rescues diosgenin-mediated anticancer activity. (a) The cell viability was tested by MTT assay in breast cancer cells treated with 50 *μΜ* diosgenin and Skp2 cDNA plasmid. (b) The cell apoptosis was tested by Annexin V-FITC/PI staining assay in breast cancer cells treated with 50 *μΜ* diosgenin and Skp2 cDNA plasmid. (c) The cell invasive activity was tested by Transwell invasion assay in breast cancer cells treated with 50 *μΜ* diosgenin and Skp2 cDNA plasmid. ∗*p* < 0.05 vs. the control group; ^#^*p* < 0.05 vs. diosgenin alone or Skp2 cDNA transfection alone.

**Figure 4:**
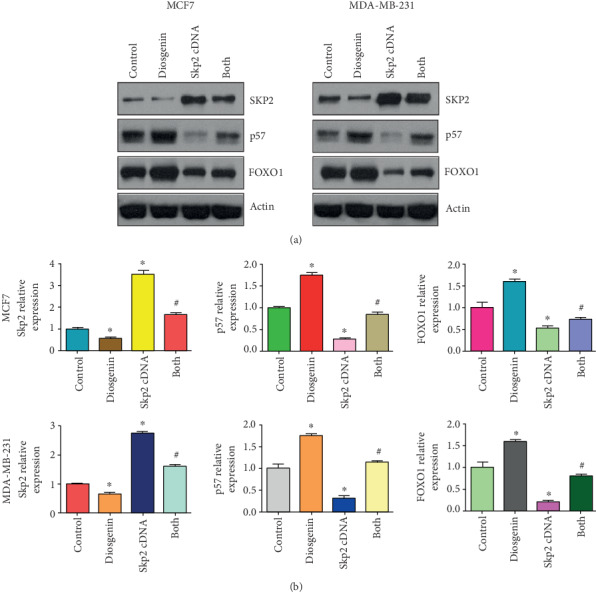
Skp2 upregulation abrogates diosgenin-mediated upregulation of p57 and FOXO1. (a) The expression of Skp2, p57, and FOXO1 was tested by western blotting analysis in breast cancer cells treated with 50 *μΜ* diosgenin and Skp2 cDNA plasmid. (b) Quantification data for (a). ∗*p* < 0.05 vs. the control group; ^#^*p* < 0.05 vs. diosgenin alone or Skp2 cDNA transfection alone.

To test whether diosgenin performed anticancer activity via regulation of the Skp2 signaling pathway in breast cancer cells, we conducted the rescue experiment to observe if overexpression of Skp2 would abrogate diosgenin-induced antitumor function. Our MTT data showed that upregulation of Skp2 increased cell viability in both MCF-7 and MDA-MB-231 cells (Figure 3(a)). Importantly, Skp2 upregulation rescued inhibition of cell viability induced by diosgenin in both breast cancer cell lines (Figure 3(a)). Moreover, upregulation of Skp2 inhibited cell apoptotic death and abrogated induction of cell apoptosis by diosgenin in two breast cancer cell lines (Figure 3(b)). Furthermore, overexpression of Skp2 promoted cell invasion in breast cancer cells, which also rescued cell invasion suppression that was mediated by diosgenin treatments (Figure 3(c)). Mechanistically, our western blotting results showed that overexpression of Skp2 abrogated the upregulation of p57 and FOXO1 that was triggered by diosgenin exposures (Figures 4(a) and 4(b)). Altogether, diosgenin mediated anticancer activity in part via suppression of Skp2 expression in breast cancer cells.

### 3.6. Downregulation of Skp2 Promoted Diosgenin-Mediated Antitumor Function

**Figure 5:**
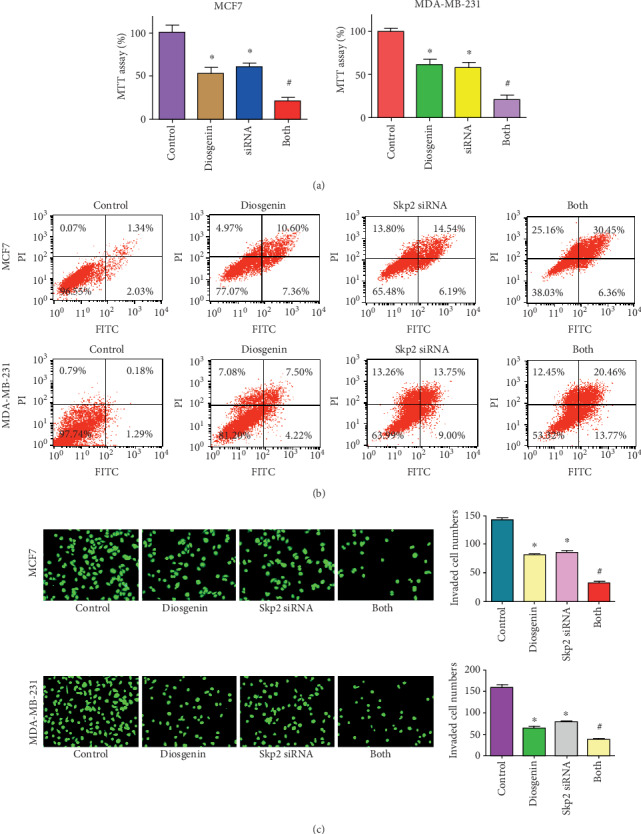
Skp2 inhibition promotes diosgenin-induced antitumor activity. (a) The cell viability was tested by MTT assay in breast cancer cells treated with 50 *μΜ* diosgenin and Skp2 siRNA. (b) The cell apoptosis was tested by Annexin V-FITC/PI staining assay in breast cancer cells treated with 50 *μΜ* diosgenin and Skp2 siRNA. (c) The cell invasive activity was tested by Transwell invasion assay in breast cancer cells treated with 50 *μΜ* diosgenin and Skp2 siRNA plasmid. ∗*p* < 0.05 vs. the control group; ^#^*p* < 0.05 vs. diosgenin alone or Skp2 siRNA transfection alone.

**Figure 6:**
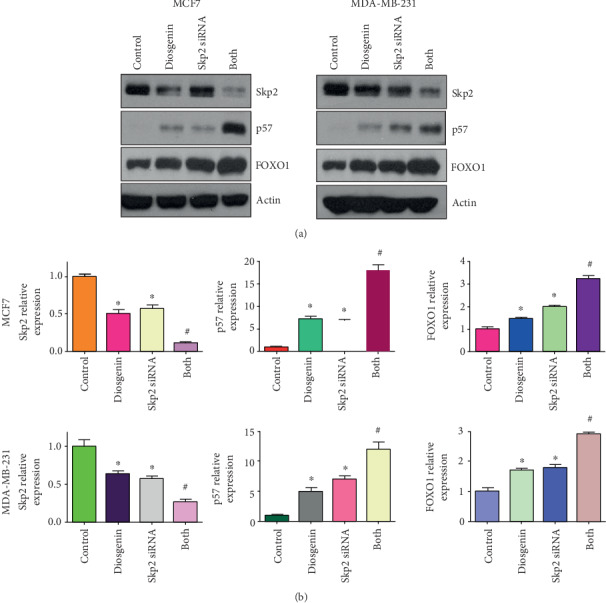
Skp2 siRNA increases diosgenin-mediated upregulation of p57 and FOXO1. (a) The expression of Skp2, p57, and FOXO1 was tested by western blotting analysis in breast cancer cells treated with 50 *μΜ* diosgenin and Skp2 siRNA. (b) Quantification data for (a). ∗*p* < 0.05 vs. the control group; ^#^*p* < 0.05 vs. diosgenin alone or Skp2 siRNA transfection alone.

To in-depth explore whether diosgenin exerts antitumor activity via modulation of Skp2 in breast cancer cells, we used Skp2 siRNA to reduce the Skp2 expression in breast cancer cells followed by diosgenin exposures for different times. Our MTT results revealed that Skp2 downregulation inhibited cell viability in MCF-7 and MDA-MB-231 cells (Figure 5(a)). Inhibition of Skp2 promoted suppression of cell viability that was triggered by diosgenin (Figure 5(a)). Moreover, Skp2 downregulation stimulated cell apoptotic death in both breast cancer cell lines, which promoted diosgenin-mediated induction of apoptosis (Figure 5(b)). Furthermore, reduction of Skp2 decreased cell invasive activity in two breast cancer cell lines, which further enhanced reduction of cell invasion mediated by diosgenin (Figure 5(c)). Our western blotting data demonstrated that Skp2 downregulation increased the expression of p57 and FOXO1 in MCF-7 and MDA-MB-231 cells (Figures 6(a) and 6(b)). The upregulation of p57 and FOXO1 by diosgenin was further increased by Skp2 siRNA transfection in breast cancer cells (Figures 6(a) and 6(b)). These data indicated that diosgenin might reduce the expression of Skp2, leading to anticancer activity in breast cancer cells.

## 4. Discussion

In the present study, we observed that diosgenin inhibited proliferation of breast cancer cells and stimulated cell apoptosis in both breast cancer cell lines. Moreover, diosgenin reduced cell invasive activity in breast cancer cells. Mechanistically, we found that diosgenin decreased the expression of Skp2 at mRNA and protein levels in breast cancer cells. Importantly, overexpression of Skp2 rescued diosgenin-induced inhibition of cell proliferation and invasion. In line with this, downregulation of Skp2 enhanced diosgenin-mediated antitumor function. Altogether, diosgenin exerts its anticancer function via suppression of Skp2 in breast cancer.

Skp2 oncoprotein has been characterized to participate in carcinogenesis, including breast cancer [9]. It has been known that Skp2 targets multiple substrates for ubiquitination and degradation, such as p27, Tob1, p21, p57, and FOXO1. Evidence has demonstrated that diosgenin has anticarcinogenic activity through reduction of lipid peroxidation via induction of antioxidant defense system in breast cancer that was induced by methyl-N-nitrosourea [14]. One study showed that diosgenin suppressed the phosphorylation of Vav2 and activation of Cdc42, leading to attenuation of cell migration of breast cancer cells, indicating that diosgenin has a therapeutic potential for metastasis therapy [15]. Diosgenin has also been reported to inhibit breast cancer stem-like cells through suppression of the Wnt/*β*-catenin signaling pathway via targeting sFRP4 (secreted frizzled-related protein 4), leading to attenuation of EMT and invasion in breast cancer [16]. Recently, one research group identified that diosgenin promoted apoptosis and triggered cell cycle arrest at the G2/M phase through regulation of Chk1 kinase, Cdc25c pathway, and Bcl-2 in breast cancer cells [17]. More importantly, diosgenin did not have effects on the growth of MCF-10A normal epithelial cells [7]. In line with this, we also observed that diosgenin did not inhibit the cell growth and Skp2 expression in MCF-10A cells (data not shown). In our study, we found that diosgenin decreased the expression of Skp2 in breast cancer cells, resulting in attenuation of breast cancer cell proliferation and motility.

Skp2 inhibitors, such as SZL-P1-41 [18], have been discovered and multiple natural compounds have been uncovered to be potential inhibitors of Skp2, including curcumin [19–21], rottlerin [13, 22], quercetin [23], and lycopene [23]. Recently, dioscin was identified as a promising inhibitor of Skp2 in colorectal cancer [24, 25]. In the current study, our data suggest that diosgenin is a potential inhibitor of Skp2 in breast cancer. Diosgenin inhibited both mRNA and protein levels of Skp2 in breast cancer cells. It is required to investigate whether diosgenin could bind with Skp2 gene promoter in the future. In addition, Skp2 has been reported to induce invasion via RhoA and facilitate EMT process via stabilizing Twist [8]. Therefore, it is essential whether diosgenin could decrease the expression of RhoA and stabilize Twist level due to Skp2 downregulation in breast cancer cells. Several groups have modified diosgenin compound to achieve better outcome in cancer patients. For example, diosgenin functionalized iron oxide magnetic nanomedicine (IONPs-D) has been developed and exhibited superior anticancer function compared with diosgenin alone in breast cancer [26]. IONPs-D exhibited antiproliferative activity, inhibited migration, and promoted apoptosis against breast cancer [26]. Another group developed novel 3-O-tethered triazoles of diosgenin and found that this compound had stronger antiproliferative activity in multiple cancer cell lines, including HBL-100 breast cancer cells [27]. Cai et al. designed and synthesized a methotrexate- (MTX-) diosgenin conjugate, which showed much more potency against MTX-resistant breast cancer cells [28]. This study indicated that MTX-diosgenin conjugation might be effective for overcoming drug resistance in breast cancer [28]. One study has shown that injection of diosgenin (10 mg/kg body weight) in nude mice with MCF-7 and MDA-MB-231 xenografts reduced tumor growth, suggesting that diosgenin can be used for treating breast cancer in mice [7]. One limitation of this study is the lack of in vivo experiment to define whether diosgenin retards tumor growth via reduction of the Skp2 level in mice.

## 5. Conclusion

In summary, diosgenin inhibited cell viability and motility via suppression of Skp2 in breast cancer cells. We concluded that diosgenin and its analogues might be useful agents in breast cancer therapy.

## Data Availability

The data used to support the findings of this study are available from the corresponding author upon request.
